# Maternal Continuing Folic Acid Supplementation after the First Trimester of Pregnancy Increased the Risk of Large-for-Gestational-Age Birth: A Population-Based Birth Cohort Study

**DOI:** 10.3390/nu8080493

**Published:** 2016-08-15

**Authors:** Sufang Wang, Xing Ge, Beibei Zhu, Yujie Xuan, Kun Huang, Erigene Rutayisire, Leijing Mao, Sanhuan Huang, Shuangqin Yan, Fangbiao Tao

**Affiliations:** 1 Department of Maternal, Child & Adolescent Health, School of Public Health, Anhui Medical University, Hefei 230032, China; wangsufangdev@126.com (S.W.); shjgexing@sina.com (X.G.); a513275541@126.com (B.Z.); xuanyujiexyj@126.com(Y.X.); wuweihk8028@136.com (K.H.); rerigene@yahoo.com (E.R.); echocrane@126.com (L.M.); sansan2009.happy@163.com (S.H.); 2 Anhui Provincial Key Laboratory of Population Health & Aristogenics, Anhui Medical University, Hefei 230032, China; 3 Ma’Anshan Maternal and Child Health Care Center, Ma’Anshan 234000, China; yanshuangqin@126.com

**Keywords:** folic acid, supplementation, pregnancy, large for gestational age, China

## Abstract

Supplementation with folic acid (FA) was proven to prevent neural tube defects (NTDs) and was recommended worldwide before and during early pregnancy. However, much less is known regarding the role of FA after the 12th gestational week (GW). This study aimed to investigate the related effects of continued FA supplementation after the first trimester of pregnancy on fetal growth. The study subjects came from the Ma’anshan-Anhui Birth Cohort Study (MABC) that recruited 3474 pregnant women from the city of Ma’anshan in Anhui Province in China during the period of May 2013 to September 2014. The information on use of vitamin and mineral supplements was recorded in different periods (the first/second/third trimester of pregnancy). Small-for-gestational-age (SGA) births were live-born infants that were <10th percentile of birth weight, and large-for-gestational-age (LGA) births were live-born infants that were ≥90th percentile of birth weight according to nomograms based on gender and gestational age from the latest standards. We used multivariable logistic regression to evaluate the effects of FA supplement consumption in the second/third trimester of pregnancy on the risk of LGA and SGA. In addition, propensity score analysis was also performed to examine the effects. In this prospective birth cohort study conducted in Chinese women who had taken FA in the first trimester of pregnancy, we found that continued FA supplementation with 400 micrograms/day in the second and third trimesters of pregnancy significantly increased the risk of LGA (RR = 1.98 (1.29, 3.04)). This relation was strong or monotonic after adjusting for maternal age, newborn’s gender, maternal pre-pregnancy BMI, maternal education level, smoking, alcohol consumption and calcium supplementation. We did not observe that continuing FA supplementation after the first trimester of pregnancy remarkably decreased the risk of SGA. The propensity score analysis showed similar results. To confirm these findings, additional investigations or trials with a large sample and the tracking of folate status throughout pregnancy are recommended.

## 1. Introduction

Folate acts as a carrier in several one-carbon unit transfers and plays a crucial role in DNA synthesis, repair and methylation, which are very important for rapid cell division and growth which occurs during pregnancy and infancy [1]. It was previously observed that pregnant women had a five- to 10-fold higher folate requirement than non-pregnant women, which increased the risk of folate deficiency among pregnant women when compared with non-pregnant women [2].

Folic acid (FA, pteroylglutamic acid), the synthetic form of folate, provides a highly stable and active form and is used widely for supplementation and food fortification. Conclusive evidence highlighted that periconceptional FA supplementation substantially reduced the risk of neural tube defects (NTDs) in offspring [3]. Such evidence has led to a clear recommendation for women who are planning a pregnancy to take 400 µg FA/day from preconception until the end of the first trimester [4]. Thus, maternal FA supplementation before and in early pregnancy is predominantly focused on preventing NTDs. Specifically, the benefits of continued FA supplementation in later pregnancy are uncertain. Currently, there are no consistent agreements on the need to maintain high levels of FA intake during the whole period of pregnancy.

In fact, folate is critically required for fetal, placental, and maternal tissue growth during pregnancy. Normally, folate required for growth reaches the maximal level in the last trimester of pregnancy [5], due to the rapid growth of the fetus and the uteroplacental system and the fetal accumulation of folate stores. Several studies revealed that when women stopped supplementations of FA or multivitamins containing FA after the first trimester of pregnancy, maternal serum and red blood cell folate concentrations decreased significantly [6,7]. The decline in maternal folate status during pregnancy is generally explained by an increase in requirements for folate associated with the growth of fetal, placental, and maternal tissue [5]. Interestingly, a recent randomized trial indicated that continuing FA supplementation at a dose of 400 µg/day after the first trimester of pregnancy produced a significant increase in maternal red blood cell folate and cord blood folate concentrations and prevented the decline in maternal serum folate [7]. Plasma homocysteine concentrations are known to be 50%–60% lower in pregnant than non-pregnant women [8] and tend to increase in later pregnancy [9]. Previous studies reported that the expected increase in homocysteine that otherwise occurred in later pregnancy might have been prevented by continuing FA supplementation in the second and third trimesters [7,9]. Continuing FA supplementation after the first trimester of pregnancy can prevent the decline in both serum folate and red blood cell folate concentrations and the increase in plasma homocysteine concentrations in the later stages of pregnancy. Additional evidence is needed to confirm whether these effects have benefits for pregnancy outcomes and child health.

In early pregnancy, there is universal agreement regarding FA supplementation, and pregnant women get consistent advice from health care professionals. However, after the 12th week of pregnancy, no official FA recommendations exist and the advice to pregnant women tends to be varied and inconsistent. Therefore, the aim of this study is to investigate the effect of continuing FA supplementation after the first trimester of pregnancy on fetal development.

## 2. Research Design and Methods

### 2.1. Cohort Study

The Ma’anshan-Anhui Birth Cohort Study (MABC) is a prospective population-based cohort study, which was designed to investigate the effects of prenatal exposure on adverse pregnant outcomes, child health and development. Pregnant women were consecutively recruited from antenatal clinics of the Maternal and Child Health (MCH) Care Center in the city of Ma’anshan of Anhui Province in China from May 2013 to September 2014, when they came to the Center for their first prenatal examination in early pregnancy. About 80% of all pregnant women living in the city come to the center for maternal and child healthcare. A total of 3474 pregnant women were recruited for this cohort. Data were collected via a face-to-face interview by trained investigators in the first/second/third trimester of pregnancy, respectively.

**Figure 1:**
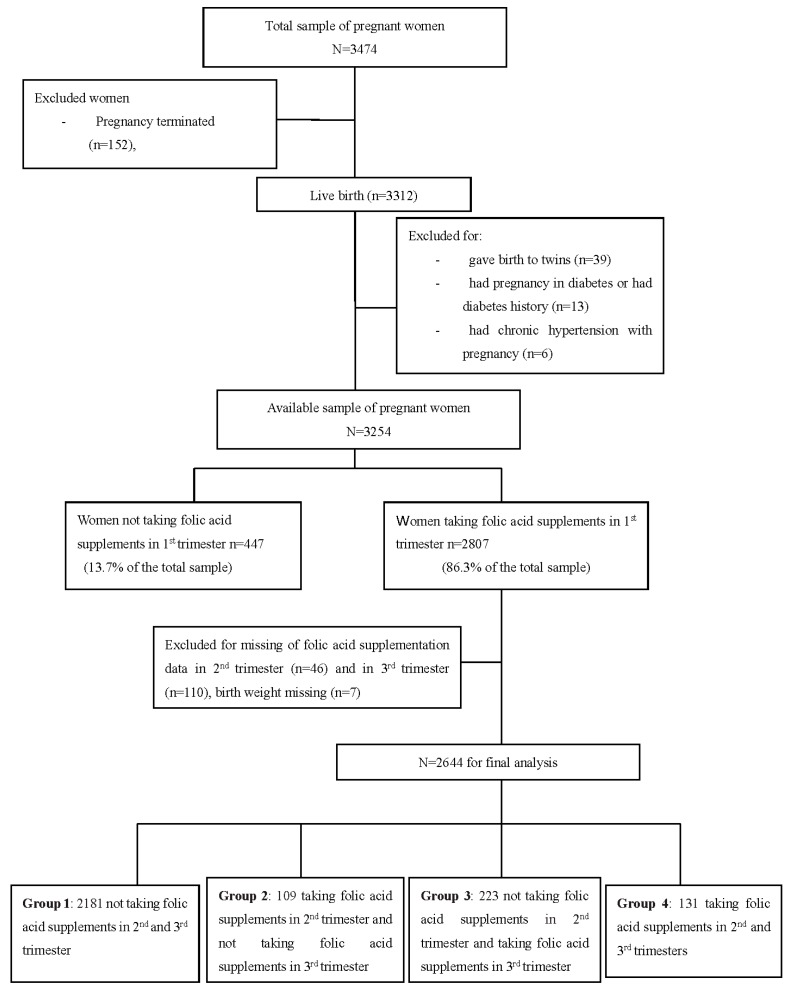
Flow diagram of recruitment and follow-up in this birth cohort study.

For this study, eligible participants were mother-and-singleton offspring pairs in which mothers had detailed information of FA and other micronutrients supplementations before and during pregnancy and offspring had detailed birth records. In our sample, 39 pregnant women giving birth to twins, 152 pregnancies terminated, 10 stillbirths, 13 having pregnancy in diabetes or having diabetes history, six having chronic hypertension with pregnancy, and 447 not taking FA supplements in the first trimester were excluded from the study. In addition, 46 pregnant women without FA supplementation information in the second trimester, 110 without FA supplementation information in the third trimester, and seven newborns with birth weight missing were also excluded. A total of 2644 mother-and-singleton offspring pairs were eligible for this study. According to maternal FA supplementation information in trimesters 2 and 3 of pregnancy, 2644 mother-and-singleton offspring pairs were divided into four groups. Group 1 included 2188 pairs, in which mothers did not take FA supplements in trimesters 2 and 3 of pregnancy; Group 2 included 109 pairs, in which mothers took FA supplements in trimester 2 and did not took FA supplements in trimester 3; Group 3 included 223 pairs, in which mothers did not take FA supplements in trimester 2 and took FA supplements in trimester 3; while Group 4 included 131 pairs, in which mothers took FA supplements in trimesters 2 and 3 of pregnancy (Figure 1). The present study was approved by the ethics committee of Anhui Medical University (Ethical approval code: 20131195). Oral and written consents were obtained from all pregnant women.

### 2.2. Folic Acid Supplementation and Pregnancy Outcomes

The information on use of FA supplements was assessed by asking if they have used these supplements in different periods (one month before pregnancy, the first/second/third trimester of pregnancy) (e.g., Have you taken FA or multivitamins containing FA in the early pregnancy?). Information regarding the date supplement use began, the frequency of use (times/week), dose and brand names of supplements was also collected. FA supplements users took FA at a dose of 400 µg/time in the pregnancy. Women who took FA supplements >two times/week during the studied period were defined as FA supplements users.

Anthropometric parameters at birth were obtained from the hospital delivery logs. Birth weight, birth length, head circumference and chest circumference of all neonates were measured by midwife at birth. Gestational age was calculated based on the last menstruation period or ultrasound to estimate if their menstruation period were irregular.

In this study, small-for-gestational-age (SGA) births were live-born infants that were <10th percentile of birth weight, and large-for-gestational-age (LGA) births were live-born infants that were ≥90th percentile of birth weight according to nomograms based on gender and gestational age from the latest standard [10]. LBW births were live-born infants that were <2500 g for birth weight, and HBW births were live-born infants that were ≥4000 g for birth weight.

### 2.3. Covariates

Extensive data were collected using a structured self-report questionnaire supervised by trained interviewers. Detailed data included maternal age, race, parity, education level, social-economic status, place of residence, having pregnancy in diabetes or having diabetes history, gestational diabetes mellitus (GDM), having chronic hypertension with pregnancy, pregnancy hypertension, smoking, alcohol consumption, and anthropometric measures, etc. In addition, paternal age, education level, smoking, alcohol consumption, anthropometric measures were also recorded.

Maternal age and body mass index (BMI) were used as continuous variables in the statistical models, except when they were used to describe the population characteristics. Maternal parity status was defined as primiparous or multiparous. Pre-pregnancy BMI was calculated from self-reported weight and height measured before conception. GDM was defined according to the American Diabetes Association (ADA) [11], and gestational hypertension was defined as systolic pressure ≥140 mmHg or diastolic pressure ≥90 mmHg after gestation. Smoking during the survey or stopping smoking at any stage of pregnancy were both defined as maternal smoking in pregnancy. Maternal drinking were defined as drinking ≥one time/week.

Among 2644 pregnant women, only six had been smoking when they were surveyed in early pregnancy, and 68 pregnant women stopped smoking when they found themselves pregnant. Only three pregnant women drank ≥one time/week. Smoking and alcohol consumption were adjusted as confounders.

### 2.4. Statistical Analysis

Data analyses were performed using the Statistical Package for the Social Sciences software (version 10.0; SPSS UK Ltd., Surrey, UK) and STATA (version 10.0). For continuous variables, ANOVA were used to determine differences among different groups and Student *t* test was used to determine differences between two groups. For categorical variables, differences were examined by using chi-square test. In order to adjust for pre-pregnancy BMI, maternal age, newborn’s sex and average monthly income, etc., a multivariable logistic regression model was used to estimate relative risk (RR) with 95% confidence intervals (95% CI) with respect to LGA and SGA incidence. In addition, given that the possible selective bias in receiving FA supplements, we performed a propensity score analysis to adjust for those factors related to selecting FA use in the second and third trimesters of pregnancy. Propensity scores were calculated using logistic regression with FA supplementation as the outcome variable and all available variables as predictors. Weighted logistic regression using the inverse of the propensity scores as weights to assess the effect of FA supplementation on risk of SGA and LGA was conducted.

All quantified data were expressed as means ± SD. All statistical tests were performed at the two-sided 0.05 level of significance.

## 3. Results

Of the 2644 pregnant women who took FA supplements in the first trimester of pregnancy, 131 (4.95%) continued FA supplementation during the second and third trimesters (Group 4). Among the pregnant women who took FA supplements in the first trimester, 2188 (82.75%) did not continue to take FA supplements after the first trimester of pregnancy (Group 1), 109 (4.12%) continued FA supplementation in the second trimester but did not use it in the third trimester (Group 2), and 223 (8.43%) did not take FA supplements in the middle of pregnancy but they did in late pregnancy (Group 3) (Figure 1).

Women who continued FA supplementation in the second and third trimesters of pregnancy were more likely to be older, living in urban areas, had higher educational levels and their husbands were also older and had higher educational levels compared with FA non-users in the same period. There were no significant differences in maternal ethnicity, pre-pregnancy BMI, parity, family per capita income, smoking status, the prevalence of GDM and pregnancy hypertension among those who continued taking FA in their second and third trimesters of pregnancy compared with those who not (Table 1).

In general, continuing FA supplementation after the first trimester was associated with higher birth weight, length, head circumference, and chest circumference compared with non-users (Table 2). The gestational weeks before delivery were similar among FA non-users (39.5 w), second trimester FA users (39.6 w), third trimester FA users (39.6 w), and FA users throughout the second and third trimesters (39.4 w).

Among 2644 mother-and-singleton offspring pairs, the overall prevalence of SGA and LGA births was 9.5% and 16.5%, respectively, and the prevalence of LBW and HBW was 1.9% and 7.9%, respectively. Among continued FA users in the second and third trimesters, the prevalence of LGA birth (26.0%) was higher than that among non-users (15.7%), second trimester FA users (15.6%) or third trimester FA users (18.8%). The prevalence of SGA birth was similar among non-users (10.0%), second trimester FA users (7.6%), third trimester FA users (5.5%), and second and third trimester FA users (9.2%). There were no significant differences in the prevalence of LBW and HBW in the four groups of our sample (Table 2).

Compared with non-users, continuing FA users in the second and third trimesters had a significantly higher risk of LGA birth (RR = 1.88; 95% CI 1.25–2.83) in the crude model. After adjusting for maternal age, infant’s sex, maternal pre-pregnancy BMI, parental education level, family monthly income per capita, area of residence, maternal parity, calcium supplementation, smoking status, alcohol consumption, GDM and gestational hypertension, a significant increased risk of LGA birth remained (adjusted RR 1.98; 95% CI 1.29, 3.04). Compared with non-users, continuing FA supplementation in the second and third trimesters did not remarkably decrease the risk of SGA (Table 3). The propensity score analysis showed similar results (shown in Table 4).

## 4. Discussion

This was a prospective birth cohort study conducted in women who had taken FA in the first trimester of pregnancy. Our findings showed that continuing FA supplementation at a dose of 400 µg/day in the second and third trimesters significantly increased the risk of LGA in Chinese women, and this relation was strong or monotonic even after adjusting for known relevant confounders. To our knowledge, this is the first study reporting that continued FA supplementation after the 12th week of pregnancy significantly increases the risk of LGA. Our results support the hypothesis that continued supplementation with FA after the first trimester is associated with higher birth weight, length, head circumference, and chest circumference. However, it did not significantly decrease the risk of SGA.

There are limited data about the association of continuing FA supplementation after the first trimester of pregnancy and birth outcomes. A systematic review and meta-analysis reported that increased folate intake after the first trimester was associated with higher birth weight and a two-fold increase in folate intake corresponded to a 2% higher birth weight [12], yet the majority of studies included in the meta-analysis were conducted at least 30 years ago and according to current standards, all of them had high risk of bias. It was observed that women with a low daily folate intake (≤240 µg/day) at gestation week 28 had an approximately two-fold greater risk of low birth weight [13]. A recent random control trial including a total of 119 women (60 women in the placebo group, 59 women in the treatment group) showed no effect of continued FA supplementation at a dose of 400 µg/day after the first trimester of pregnancy on birth weight, and this finding may be explained by the relatively small sample size and, thus, potentially insufficient statistical power to observe a small effect on birth weight [7,14]. The relative paucity of the collected reports suggested an urgent need to develop further high quality studies focusing on health outcomes of continuing folate supplementation after the first trimester.

The exact biological mechanisms explaining why continuing FA supplementation increased the risk of LGA remain poorly understood. Folate plays critical roles in nucleotide (purine and thymidine) synthesis, which can subsequently affect DNA synthesis and mitotic cell division [15]. During pregnancy, fetal growth causes an increase in the total number of rapidly dividing cells, which leads to increased requirements for folate [2]. It has been observed that, with inadequate folate intake, maternal plasma and red blood cell (RBC) folate concentrations decrease from the fifth month of pregnancy until several weeks after delivery [16] and their plasma homocysteine concentrations increase in later pregnancy [7,9]. There are several reasons that continuing FA supplementation after the first trimester of pregnancy may increase the risk of LGA. Firstly, continued FA supplementation at a dose of 400 µg/day in the second and third trimesters significantly increased maternal red blood cell folate and cord blood folate concentrations and prevented the decline in serum folate and elevation in plasma homocysteine concentrations [7], which may be beneficial to fetal growth. FA is an important methyl-group vitamin, and maternal plasma FA levels are associated with offspring DNA methylation [17,18,19], and the latter is in relation to fetal development and birth weight [20,21]. It was reported that methylating micronutrient supplementation (folate, choline, vitamins B6, and B12) during pregnancy increased fetal weight by influencing gene expression and IGF signaling of fetal liver and skeletal muscle in Pietrain gilts [22]. Secondly, maternal FA supplementation in the second trimester reduced the risk of preeclampsia by improving placental and systemic endothelial functions [23], while maternal preeclampsia is very adverse for fetal development. Thirdly, prenatal micronutrient supplements cumulatively increase fetal growth, and each unit of cumulative micronutrient intake was associated with a 1.6 g ((95% CI: 0.3, 3.1); *p* = 0.019) higher birth weight [24]. Continuing FA supplementation after the first trimester of pregnancy increased cumulative FA intake, which was probably related to a higher birth weight. However, in the Ma’anshan-Anhui Birth Cohort Study, we did not observe that periconceptional FA supplementation was associated with birth weight (data not shown). The possible reason is that nutrients in the first trimester may be more important for development and differentiation of various organs, and later in pregnancy they may be important for overall fetal growth [25]. Taken together, it is plausible that use of FA or a multivitamin containing FA throughout the whole pregnancy increases the LGA risk by its actions on placental and fetal growth.

In this study, we did not find that continued FA supplementation after the 12th week of pregnancy significantly decreased the risk of SGA. First of all, it must be taken into account that the potential reason for the non-significant results of continuing FA use after the first trimester on SGA may be explained by the effect of inadequate sample size, as, among 2644 pregnant women who took FA supplements in early pregnancy in our sample, only 131 continued FA supplementation throughout the whole pregnancy. Secondly, it was related to the high folate status at baseline in our sample as a result of the study design, which excluded any woman who did not take FA during the first trimester. Moreover, with the development of economy, Chinese diets are rich in various foods and our population is generally replete with folate. We once chose 721 pregnant women from the cohort and measured their serum folate concentrations in early pregnancy. The mean level was 51.4 ± 15.6 nmol/L, and the minimum value was 27.9 nmol/L (data not shown). It was observed that higher maternal folate concentrations during late pregnancy had no benefit for reducing the risk of SGA in a multiethnic Asian population [26]. If there is an effect of FA supplementation during pregnancy on SGA, it may possibly only be observed in the face of more generalized maternal folate depletion [27].

In the present study, there were no significant differences in the prevalence of LBW and HBW in the four groups of our sample. Firstly, due to the small sample size and lower prevalence of LBW and HBW (compared with LGA), there were fewer babies of LBW and HBW in Group 2, Group 3, and Group 4, which might lead to the non-significant results of continuing FA supplementation after the first trimester on the risk of LBW and HBW. However, there was also one possibility that continuing FA supplementation at a dose of 400 µg/day in the second or third trimester of pregnancy could promote fetal growth moderately, but it did not increase the risk of HBW, i.e., it did not produce heavy babies, which was similar to our results. It was reported that longer birth length, higher birth weight, or larger head circumferences within the normal birth size range were associated with higher IQ scores in Asian children [28]. The Hertfordshire Cohort Study found that death rates from cardiovascular disease fell progressively between those with low and high birth weights, and the relationship was graded among women and men [29]. This trend did not depend on differences in the length of gestation and therefore reflected differences in fetal growth rates [29]. In this sense, continuing FA supplementation after the first trimester may be beneficial to the offspring, and being LGA could be an advantage for the baby, however not for the mother who is giving birth, for there are disadvantages of a more difficult birth and other related obstetrical problems.

Our study has several strengths. To the best of our knowledge, it is the first report that continuing FA supplementation after the 12th week of pregnancy significantly increased the risk of LGA. In addition, it is a prospective birth cohort study with a high level of evidence to show causal inference. Furthermore, we adjusted for known relevant confounders. Likewise, our study has limitations worth noting. First, no dietary information was collected and we could not evaluate the effects of the dietary folate intake. Second, our sample size was relatively small. Third, due to small sample size among FA users in the 2nd and 3rd trimesters, we did not conduct a subgroup analysis and the effects of other vitamins were not excluded.

In conclusion, in this prospective cohort of Chinese women, we found that continuing FA supplementation in the second and third trimesters promoted fetal growth and significantly increased the risk of LGA, yet it did not reduce the risk of SGA. FA supplementation during pregnancy is recommended worldwide during preconception and the first trimester to prevent neural tube defects. Our findings suggested the potential effects of FA supplementation in the second and third trimesters of pregnancy on birth outcomes. Additional studies with a large sample size and the tracking of folate status throughout pregnancy are required to confirm these findings.

## Figures and Tables

**Table 1:** Parental characteristics according to FA supplement use after the first trimester of pregnancy.

Parental Characteristics	Group 1	Group 2	Group 3	Group 4	*p*
	*n* = 2181	*n* = 109	*n* = 223	*n* = 131	
**Maternal characteristics**					
Age (*y*, *means ± SD*)	26.3 ± 3.4	27.0 ± 3.9	26.5 ± 3.2	28.3 ± 4.1	0
≤24 (*n* (*%*))	733 (33.6)	30 (27.5)	58 (26.0)	20 (15.3)	
25–29 (*n* (*%*))	1122 (51.4)	59 (54.1)	134 (60.1)	66 (50.4)	0
≥30 (*n* (*%*))	326 (14.9)	20 (18.3)	31 (13.9)	45 (34.4)	
Ethnicity (Han) (%)	98.4	95.4	97.3	99.2	0.127
Residence (*n* (*%*))					
Urban	1714 (78.7)	92 (86.0)	185 (83.3)	119 (90.8)	
Suburb	286 (13.1)	10 (9.3)	26 (11.7)	6 (4.6)	0.009
Rural	177 (8.1)	5 (4.7)	11 (5.0)	6 (4.6)	
BMI ^a^ (*kg/m^2^*, *means* ± *SD*)	20.8 ± 2.8	20.8 ± 2.5	20.3 ± 2.3	20.7 ± 3.1	0.07
<18.5(*n* (*%*))	419 (19.2)	21 (19.3)	39 (17.5)	36 (27.5)	
18.5–24.9 (*n* (*%*))	1594 (73.1)	81 (74.3)	174 (78.0)	85 (64.9)	0.134
≥25 (*n* (*%*))	168 (7.7)	7 (6.4)	10 (4.5)	10 (7.6)	
Parity (*n* (*%*))					
1	1996 (91.5)	99 (90.8)	210 (94.2)	119 (90.8)	0.512
≥2	185 (8.5)	10 (9.2)	13 (5.8)	12 (9.2)	
Educational (years)	13.5 ± 3.1	13.7 ± 2.9	13.8 ± 3.0	14.6 ± 2.9	0.003
≤9 (*n* (*%*))	378 (17.3)	13 (11.9)	27 (2.1)	14 (10.7)	
10–15 (*n* (*%*))	1182 (54.2)	65 (59.6)	128 (57.4)	63 (48.1)	0.011
>15 (*n* (*%*))	621 (28.5)	31 (28.4)	68 (30.5)	54 (41.2)	
Monthly income (*n* (*%*))					
Low income ^b^	544 (25.2)	25 (23.1)	43 (19.3)	24 (18.3)	
Middle income ^b^	935 (43.2)	48 (44.4)	111 (49.8)	55 (42.0)	0.139
High income ^b^	683 (31.6)	35 (32.4)	69 (30.9)	52 (39.7)	
Smoking (*n* (*%*))	66 (3.0)	0	4 (1.8)	4 (3.1)	0.22
GDM (*n* (*%*))	276 (12.7)	13 (11.9)	31 (13.9)	22 (16.8)	0.538
Pregnancy hypertension (*n* (*%*))	129 (5.9)	5 (4.6)	6 (2.7)	12 (9.2)	0.07
**Paternal characteristics**					
Age (*y*, *means ± SD*)	28.1 ± 4.4	29.2 ± 4.8	28.6 ± 4.7	30.3 ± 5.1	0
≤24 (*n* (*%*))	398 (18.3)	15 (13.8)	35 (15.7)	8 (6.1)	
25–29 (*n* (*%*))	1136 (52.1)	49 (45.0)	109 (48.9)	61 (46.6)	0
≥30 (*n* (*%*))	645 (29.6)	45 (41.3)	79 (35.4)	62 (47.3)	
Educational (years)					
≤9 (*n* (*%*))	304 (13.9)	11 (10.1)	18 (8.1)	3 (2.3)	
10–15 (*n* (*%*))	1206 (55.3)	59 (54.1)	126 (56.5)	63 (48.1)	0
>15 (*n* (*%*))	671 (30.8)	39 (35.8)	79 (35.4)	65 (49.6)	
BMI (*kg/m^2^*, *means* ± *SD*)	23.4 ± 3.5	23.5 ± 5.1	23.4 ± 3.4	23.5 ± 3.3	0.984

^a^ BMI before pregnancy; ^b^ Low income for <2500 RMB per capita per month; middle income for 2500–4000 RMB per month; high income for ≥4000 RMB per month.

**Table 2:** Characteristics of 2644 newborns according to FA supplementation use after the first trimester of pregnancy.

Newborn Characteristics	Group 1	Group 2	Group 3	Group 4	*p*
	*n* = 2181	*n* = 109	*n* = 223	*n* = 131	
Sex, M (%)	1114 (51.1)	57 (52.3)	99 (44.6)	76 (58)	0.098
Gestation week at labor	39.5 ± 1.2	39.6 ± 1.1	39.6 ± 1.0	39.4 ± 1.2	0.411
Birth weight (m ± SD in g)	3359.2 ± 427.6	3432.6 ± 422.4	3453.0 ± 432.4	3435.1 ± 429.8	0.002
Birth length (m ± SD in cm)	50.0 ± 1.8	50.1 ± 1.6	50.4 ± 1.6	50.3 ± 1.5	0.02
Head circumference (m ± SD in cm)	34.0 ± 1.6	34.4 ± 1.8	34.1 ± 1.4	34.3 ± 1.3	0.024
Chest circumference (m ± SD in cm)	33.5 ± 1.5	33.8 ± 1.5	33.7 ± 1.5	33.8 ± 1.4	0.019
LGA (*n* (*%*))	342 (15.7)	17 (15.6)	42 (18.8)	34 (26.0)	0.015
SGA (*n* (*%*))	217 (10.0)	6 (5.5)	17 (7.6)	12 (9.2)	0.323
LBW (*n* (*%*))	48 (2.2)	0	2 (0.9)	1 (0.9)	0.158
HBW (*n* (*%*))	165 (7.6)	12 (11.0)	24 (10.8)	9 (6.9)	0.216

m: mean, SD: standard deviation.

**Table 3:** Relative risks of LGA and SGA in pregnant women according to folic acid supplementation after early pregnancy * (relative risk (RR) and 95% confidence interval).

RRe	Group 1	Group 2	Group 3	Group 4
	*n* = 2181	*n* = 109	*n* = 223	*n* = 131
**Risk of LGA birth**				
model 1	1	0.99 (0.58, 1.69)	1.25 (0.87, 1.78)	1.88 (1.25, 2.83) **
model 2	1	0.99 (0.58, 1.68)	1.24 (0.86, 1.77)	1.85 (1.23, 2.79) **
model 3	1	0.98 (0.59, 1.70)	1.26 (0.88, 1.80)	1.97 (1.30, 2.98) **
model 4	1	1.05 (0.61, 1.80)	1.32 (0.92, 1.90)	2.07 (1.36, 3.15) **
model 5	1	1.05 (0.61, 1.80)	1.29 (0.89, 1.87)	1.98 (1.29, 3.04) **
**Risk of SGA birth**				
model 1	1	0.53 (0.23, 1.21)	0.75 (0.45, 1.25)	0.91 (0.50, 1.68)
model 2	1	0.54 (0.23, 1.24)	0.75 (0.45, 1.26)	0.99 (0.54, 1.85)
model 3	1	0.53 (0.23, 1.22)	0.74 (0.44, 1.25)	0.92 (0.50, 1.71)
model 4	1	0.51 (0.22, 1.19)	0.74 (0.44, 1.25)	0.80 (0.43, 1.51)
Model 5	1	0.55 (0.23, 1.27)	0.77 (0.45, 1.29)	0.90 (0.48, 1.70)

* Model 1 is crude RR. Model 2 adjusts for maternal age in pregnancy and the newborn’s gender; model 3 includes further adjustment for maternal BMI before pregnancy; model 4 plus maternal education level, family monthly income per capita, parity, area of residence, maternal smoking status, alcohol consumption and calcium supplementation; model 5 plus gestational diabetes mellitus and gestational hypertension; ** *p <* 0.01 compared with Group 1 (reference).

**Table 4:** Relative risks * of LGA and SGA in pregnant women according to folic acid supplementation after early pregnancy (adjusting for propensity score or not).

	RR Not Adjusting for Propensity Score	RR Adjusting for Propensity Score
**Risk of LGA birth**		
Group 1	1	1
Group 2	0.97 (0.56, 1.69)	1.05 (0.58, 1.88)
Group 3	1.32 (0.91, 1.90)	1.35 (0.94, 1.96)
Group 4	1.92 (1.25, 2.98)	1.87 (1.21, 2.87) **
**Risk of SGA birth**		
Group 1	1	1
Group 2	0.54 (0.23, 1.26)	0.50 (0.21, 1.21)
Group 3	0.70 (0.42, 1.18)	0.71 (0.42, 1.18)
Group 4	0.86 (0.45, 1.64)	0.67 (0.33, 1.32)

* (Relative risk (RR) and 95% confidence interval); ** *p <* 0.01 compared with Group 1 (reference).
